# Development of an FPW Biosensor with Low Insertion Loss and High Fabrication Yield for Detection of Carcinoembryonic Antigen

**DOI:** 10.3390/s16111729

**Published:** 2016-11-08

**Authors:** Je-Wei Lan, I-Yu Huang, Yu-Cheng Lin, Chang-Yu Lin, Jian-Lin Chen, Chia-Hsu Hsieh

**Affiliations:** 1 Department of Electrical Engineering, National Sun Yat-sen University, Kaohsiung 80424, Taiwan; d993010003@student.nsysu.edu.tw (J.-W.L.); iyuhuang@mail.nsysu.edu.tw (I-Y.H.); 2 Department of Engineering Science, National Cheng Kung University, Tainan 70101, Taiwan; yuclin@mail.ncku.edu.tw; 3 Sunonwealth Electric Machine Industry Co. Ltd., Kaohsiung 80673, Taiwan; orach3@gmail.com; 4 Powerchip Technology Corporation, Hsinchu 30078, Taiwan; niceday810@gmail.com

**Keywords:** flexural plate-wave, low insertion loss, high fabrication yield, focus-type interdigital transducers, reflective grating structure, carcinoembryonic antigen biosensor

## Abstract

In the last two decades, various flexural plate-wave (FPW)-based biosensors with low phase velocity, low operation frequency, high sensitivity, and short response time, have been developed. However, conventional FPW transducers have low fabrication yield because controlling the thickness of silicon/isolation/metal/piezoelectric multilayer floating thin-plate is difficult. Additionally, conventional FPW devices usually have high insertion loss because of wave energy dissipation to the silicon substrate or outside area of the output interdigital transducers (IDTs). These two disadvantages hinder the application of FPW devices. To reduce the high insertion loss of FPW devices, we designed two focus-type IDTs (fan-shaped and circular, respectively) that can effectively confine the launched wave energy, and adopted a focus-type silicon-grooved reflective grating structure (RGS) that can reduce the wave propagation loss. To accurately control the thickness of the silicon thin-plate and substantially improve the fabrication yield of FPW transducers, a 60 °C/27 °C two-step anisotropic wet etching process was developed. Compared with conventional FPW devices (with parallel-type IDTs and without RGS), the proposed FPW devices have lower insertion loss (36.04 dB) and higher fabrication yield (63.88%). Furthermore, by using cystamine-based self-assembled monolayer (SAM) nanotechnology, we used the improved FPW device to develop a novel FPW-based carcinoembryonic antigen (CEA) biosensor for detection of colorectal cancer, and this FPW-CEA biosensor has a low detection limit (5 ng/mL), short response time (<10 min), high sensitivity (60.16–70.06 cm^2^/g), and high sensing linearity (R-square = 0.859–0.980).

## 1. Introduction

Various acoustic microsensors have been developed for molecular mass detection in the last two decades, including thickness shear mode (TSM), surface acoustic wave (SAW), shear horizontal acoustic plate mode (SH-APM), and flexural plate-wave (FPW) [1,2,3,4,5,6,7]. Among these acoustic microsensors, FPW devices are most suitable for applications in clinical, environmental, biological, and biomedical detection because they have low phase velocity, low operation frequency, small radiation loss in the testing liquid, high sensitivity, and short response time. However, despite these advantages, FPW devices have high insertion loss (>−50 dB) and low fabrication yield (<10%), which limit their applications.

**Figure 1:**
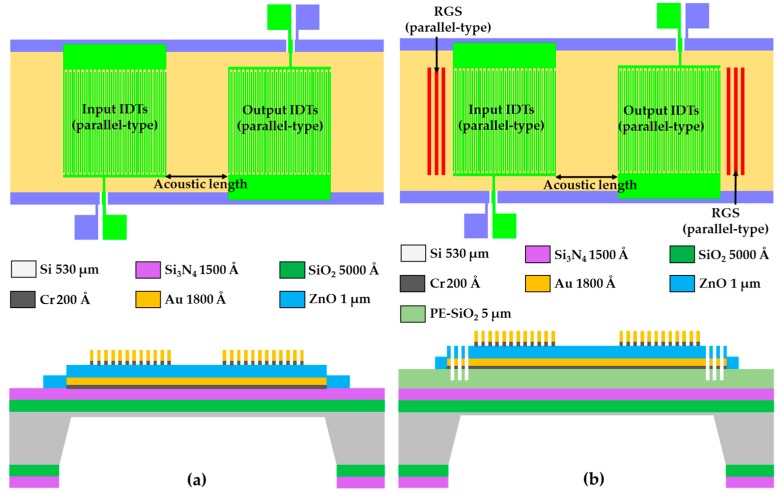
Layout diagram and cross-sectional structure of two conventional FPW transducers; (**a**) without, and (**b**) with a parallel-type Si-grooved RGS.

The two major advantages of conventional FPW devices result from their design. In a conventional FPW device [7], as shown in Figure 1a, one pair of parallel-type metal-thin-film interdigital transducers (IDTs) is usually implemented on a few μm-thick silicon/isolation/metal/piezoelectric multilayer floating thin-plate. The ultrasonic flexural plate-wave of the FPW transducer is launched and, respectively, received by the left-side input IDTs and right-side output IDTs. Some of the wave energy dissipates to the silicon substrate or the outside area of the output IDTs, increasing the insertion loss of the FPW device. Previously [7,8], we adopted a parallel-type silicon-grooved reflective grating structure (RGS), as shown in Figure 1b, to reduce the wave energy loss, which improved the insertion loss from −51.08 dB to −40.85 dB. To further enhance the propagation and reflection efficiency of the launched flexural plate-wave, reduce the insertion energy loss, and increase the signal-to-noise (S/N) ratio, we first introduced focus-type metal-thin-film IDTs and a focus-type silicon-grooved RGS into the geometric designs of the FPW device. Two innovative focus-type IDTs and RGSs are designed and compared in this study: one with a fan-shaped geometry and one with a circular geometry.

Our previous study also revealed the difficulty to accurately control the thickness of the few μm-thick silicon/isolation/metal/piezoelectric multilayer floating thin-plate of the FPW transducer during the 80–90 °C anisotropic wet etching process, in which the fabrication yield of conventional FPW microsensors is usually low (<10%). To control the thickness of the silicon thin-plate, we developed a two-step low-temperature (60 °C/27 °C) anisotropic wet etching process, which substantially improved the fabrication yield of the FPW transducer.

The improved FPW transducer with a Cr/Au/cystamine self-assembled monolayer (SAM)/glutaraldehyde/CEA antibody multilayer integrated to the backside cavity, was then developed into a novel carcinoembryonic antigen (CEA) biosensor. According to the World Cancer Report 2014 by the International Agency for Research on Cancer (IARC), the specialized cancer agency of the World Health Organization (WHO), the global number of cases of colorectal cancer increased from 1.23 million in 2008 to 1.4 million in 2012 [9,10]. Since the concentration of CEA is an important indicator of colorectal cancer, the developed CEA biosensor, which has low insertion loss and high fabrication yield, can enhance the detection of colorectal cancer.

## 2. Theory and Design

### 2.1. Theoretical Description of the FPW Device

With low phase velocity, low operation frequency, small radiation loss in the testing liquid, high sensitivity, and short response time, FPW-based microsensors are most suitable for clinical, biomedical, and environmental applications. In an FPW device, the acoustic wave propagates in a floating thin-plate (usually constructed by a silicon/isolation/metal/piezoelectric multilayer) whose thickness (approximately 3–20 μm) is smaller than the wavelength (approximately 75–120 μm) [7,8,11]. When the ratio of thin-plate thickness to wavelength is smaller than 1, the phase velocity of the FPW device is lower than the sound velocity in most liquids, so only a minor amount of energy dissipates from the thin solid plate to the testing liquid. Additionally, because lower phase velocity (*V_p_*) causes a lower operating frequency (*f*_0_) for a given wavelength, the signal readout integrated circuit (IC) of an FPW-based microsensor can be designed more easily than those of other high-*f*_0_ acoustic microsensors.

In addition, before and after the testing sample was dipped on the FPW-based microsensor, an obvious operating frequency shift (denoted as *Δf*) can be measured and further used to estimate the concentration of the detection target of the testing sample. Based on FPW theory [1,7,8,12], the operating frequency shift caused by mass-loading of the silicon/isolation/metal/piezoelectric floating thin-plate can be expressed by the following equation [1,13,14]:

(1) $$\frac{\Delta f}{f_{0}}{\;=\;S}_{m}\Delta_{m}$$

where *S_m_* denotes the mass-sensitivity of the FPW device in mass per unit area (*Δ_m_*). Since the mass of the floating thin-plate of the FPW device is much smaller than that of the bulky substrates adopted in other acoustic microsensors, any small changes in the mass can affect phase velocity and operating frequency shift (>5 kHz). Therefore, the mass-sensitivity of FPW microsensors is higher than those of other acoustic microsensors.

### 2.2. Layout Specification of the Proposed Focus-Type IDTs/RGS

**Figure 2:**
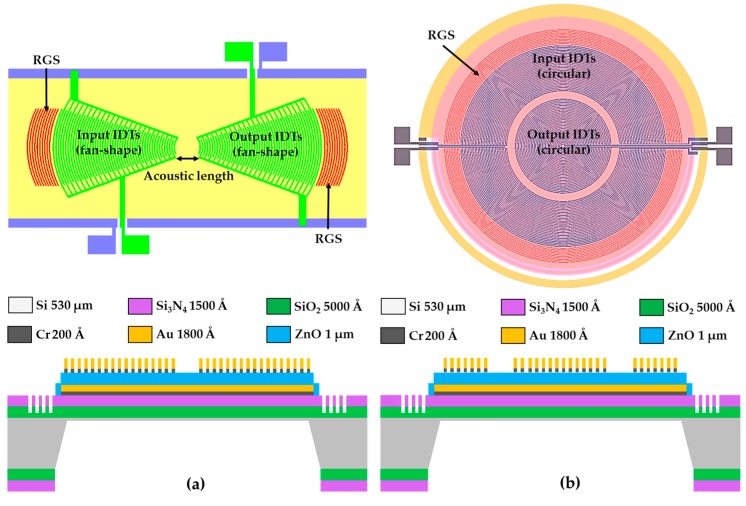
Layout diagram and cross-sectional structure of the presented FPW devices with (**a**) fan-shaped and (**b**) circular IDTs and a Si-groove RGS.

To improve the efficiency of the launched flexural plate-wave propagated from the left-side input IDTs to the right-side output IDTs, we first introduced focus-type metal-thin-film IDTs and a focus-type silicon-grooved RGS into the geometric designs of the FPW device, and then developed two innovative focus-type IDTs and RGS configurations, fan-shaped and circular IDTs/RGS, whose corresponding layout diagrams and cross-sectional structures are shown in Figure 2a,b. Unlike conventional parallel-type IDTs/RGS configurations (Figure 1), both the fan-shaped and circular IDTs/RGS are shaped as concentric circular arcs and can be used to increase wave density, beam-width compression ratio, reflection of the wave launched from the input IDTs, and wave reception by the output IDTs [15]. Consequently, the insertion energy loss can be reduced, and the signal-to-noise (S/N) ratio can be improved.

The major design parameters of the presented focus-type IDTs/RGS are listed in Table 1, and the insertion loss of the two proposed FPW devices will be compared and discussed in the subsequent section. The IDT structure is a bidirectional device and can be converted into a unidirectional device with a reflector, such as an RGS configuration [12]. Both the finger width and gap of the two focus-type IDTs and the RGS were designed to be 25 μm, so the theoretical wavelength of the FPWs generated from these IDTs is equal to 100 μm (four times of IDT finger width). Each RGS of the FPW devices was constructed from 10 pairs of etched Si_3_N_4_/SiO_2_ grooves. The input and output RGS of the FPW devices were both constructed from 10 pairs of Cr/Au fingers.

We also investigated how the area of the floating thin-plate in the FPW device affected the fabrication yield. The area of the floating thin-plate in the FPW device with the fan-shaped IDTs/RGS configuration was approximately 30 mm^2^ (6.5 mm × 4.5 mm), approximately two times of that of the FPW device with a circular IDTs/RGS configuration (3.9 mm × 3.9 mm). Under the area limitation, the maximum number of input and output IDTs’ Cr/Au finger pairs that can be designed in the two types of FPW devices are 25 and 10, respectively. The separation gap between the two proposed focus-type RGS and IDTs is 37.5 μm.

## 3. Experimental

### 3.1. Fabrication of the FPW Device

**Figure 3:**
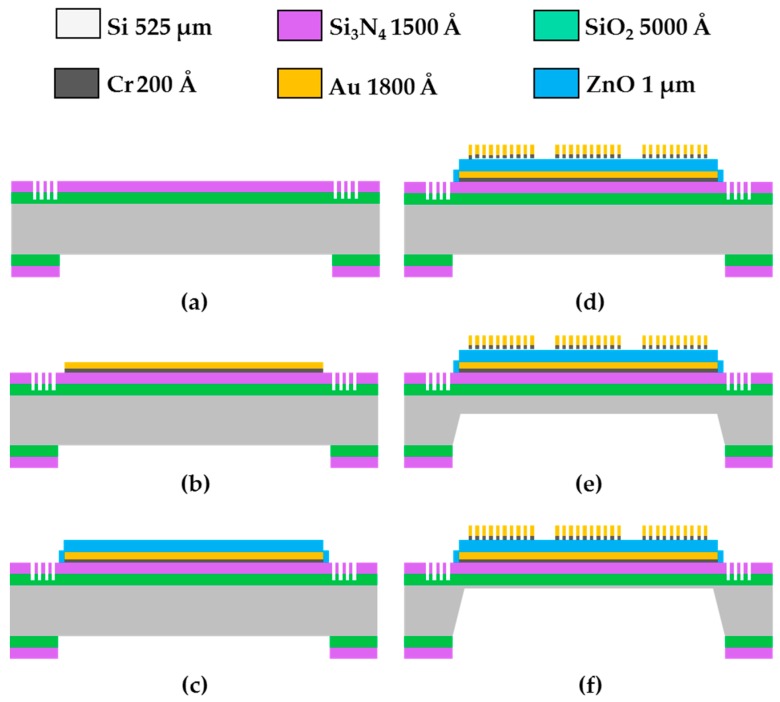
Main processing steps of the proposed FPW transducer: (**a**) 0.5/0.15-μm-thick SiO_2_/Si_3_N_4_ deposition, 0.3-μm-deep Si-groove RGS etching, and backside SiO_2_/Si_3_N_4_ patterning; (**b**) 0.02/0.15-μm-thick Cr/Au ground electrode deposition and patterning; (**c**) 1-μm-thick ZnO deposition and patterning; (**d**) 0.02/0.18-μm-thick Cr/Au IDT deposition and patterning; (**e**) anisotropic etching of the backside silicon using 30 wt % KOH at 60 °C for 35.5 h; and (**f**) 27 °C for 6.85 h.

The main processing steps of the two focus-type FPW devices were identical, and the representative structure of the FPW device with circular-type IDTs/RGS is shown in Figure 3. The 5000-Å-thick silicon dioxide and the 1500-Å-thick silicon-rich low-stress nitride were grown and deposited on a 525-μm-thick four-inch (100) silicon wafer using a thermal furnace and low-pressure chemical vapor deposition system. A 0.3-μm-deep Si_3_N_4_/SiO_2_-groove RGS configuration and a 0.65-μm-deep Si_3_N_4_/SiO_2_ backside cavity window were patterned by two photolithography processes and two reactive ion etching (RIE) processes (Figure 3a). A 200-Å-thick Cr layer and a 1500-Å-thick Au layer were continually deposited onto the Si/SiO_2_/Si_3_N_4_ layers by an e-beam evaporator to form a ground plane of the FPW device. The (111) plane of the gold (Au) metal layers well matched with the (002) plane of the piezoelectric layer (ZnO) [8,11,12]. The Au and Cr thin layers were patterned by the Au etchant (3%I_2_: 40%KI: 57%H_2_O) and the Cr etchant (Cr-7T), respectively (Figure 3b). Transduction was not affected by the patterned conducting ground plane opposite and close to the IDTs, nor did the transverse electric fields set up by the voltages between adjacent transducer fingers did affect transduction.

Many piezoelectric thin-films have been developed for the application of acoustic microsensors, such as ZnO, AlN, and Lead Zirconate Titanate (PZT) thin-films but, in our study, a high-quality C-axis-oriented 1 μm-thick ZnO piezoelectric layer was deposited on the Cr/Au ground plane by RF magnetron sputtering and patterned by wet etching (3% H_3_PO_4_: 3% CH_3_COOH: 94% H_2_O), as shown in Figure 3c. Furthermore, to construct the IDTs of the proposed FPW devices, a 200-Å-thick Cr and an 1800-Å-thick Au layer were deposited by an e-beam evaporator and patterned by a lift-off photolithographic method, as shown in Figure 3d. These processes are similar to those in our previous studies [7,8,12], but to accurately control the thickness of the silicon thin-plate and substantially improve the fabrication yield of the FPW transducer, we replaced the original 80 °C [8] or 90 °C [12] one-step high-temperature anisotropic wet etching process with a 60 °C/27 °C two-step low-temperature anisotropic wet etching process. First, most of the backside silicon substrate (approximately 475 μm thick) of the four-inch Si-wafer (525 μm thick) was quickly removed in 30 wt %, 60 °C KOH anisotropic etching solution (Figure 3e). Second, the remainder of the 50-μm-thick silicon thin-plate was slowly etched to 15 ± 2 μm using the same anisotropic wet etching solution, but at a lower temperature (27 °C), as shown in Figure 3f. The total thickness of the Si/SiO_2_/Si_3_N_4_/Cr/Au/ZnO/Cr/Au floating thin-plate of the implemented FPW device was only 16.85 μm thick. In our previous study, it manifested that a floating thin-plate less than 20 μm thick allows the center frequency and mass sensitivity of an FPW device to be controlled within 10–20 MHz and 50–100 cm^2^/g. Thus, a low operating frequency and moderate mass sensitivity are beneficial for the readout circuit design in developing an FPW-based biosensing microsystem.

### 3.2. Fabrication of the FPW-Based CEA Biosensor

To further develop an FPW-based CEA biosensor, a backside electrode of 0.02-μm-thick Cr and 0.15-μm-thick Au must be deposited on the backside silicon thin-plate before SAM immobilization. Molecular self-assembly is the spontaneous organization of molecules into stable, structurally well-defined aggregates. In this study, the cystamine material with amine-group bonds was adopted as the SAM between the backside sensing gold layer and the CEA antibody layer. Before the immobilization of the cystamine SAM layer, the backside gold layer surface of the FPW device was pretreated with “piranha” solution (70 wt % H_2_SO_4_:30 wt % H_2_O_2_) for 30 min to create a hydrophilic surface and improve the adsorption of cystamine SAM molecules. Then, the surface was washed with deionized (DI) water three times and dried at room temperature. To immobilize the SAM layer onto the gold layer, the chip was immersed in a cystamine solution (0.02 M) for 1 h, washed with DI water three times, and air-dried. The chips were further incubated in 1.25 wt % aqueous glutaraldehyde cross-linking reagent for 1 h and washed with DI water three times.

**Figure 4:**
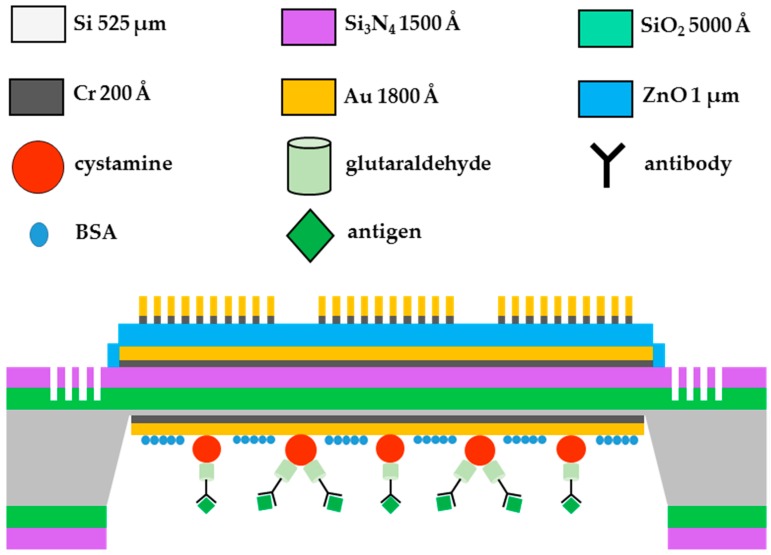
Final configuration of the proposed FPW-based CEA biosensor.

After the highly-purified mouse anti-human CEA antibody layer was coated on the surface of the backside glutaraldehyde layer, a dilute bovine serum (BSA) layer was used to block the CEA antibody-coated surface to avoid non-specific absorption. The final configurations of the FPW-based biosensor and the integrated cystamine SAM/glutaraldehyde/CEA antibody/CEA antigen multilayer are schematically shown in Figure 4, and the detailed procedure was as follows:

1. Wash three times with 1 c.c. of phosphate-buffered saline solution;
2. Dip 10 μL of diluted rabbit CEA antibody (27 °C, 2 h);
3. Inject 200 μL of Tween-20 wash buffer three times;
4. Inject 10 μL of 1 wt % BSA solution (27 °C, 0.5 h);
5. Inject 200 μL of Tween-20 wash buffer three times; and
6. Inject 10 μL of diluted human CEA antigen (27 °C, 2 h).

## 4. Results and Discussion

### 4.1. Structural Inspection and Fabrication Yield Investigation

**Figure 5:**
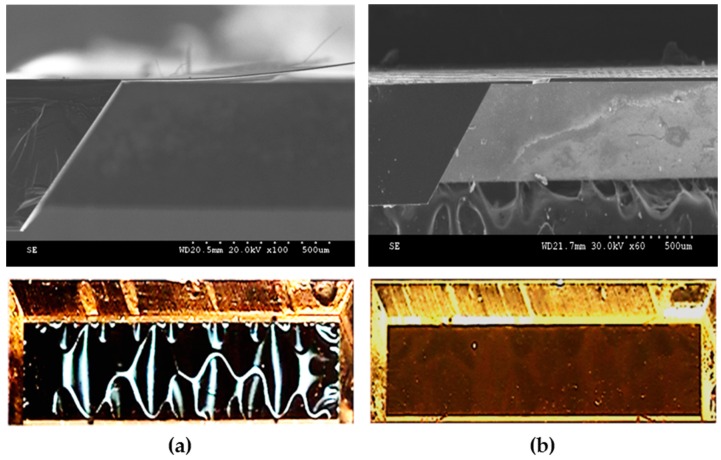
Cross-sectional SEM and bottom-view optical micrographs of the implemented FPW device using the (**a**) 80 °C and (**b**) 60 °C/27 °C two-step low-temperature anisotropic silicon etching processes.

Our previous studies have revealed that as the temperature of the anisotropic etching process increases, the silicon etching rate increases. However, controlling the thickness of the Si/SiO_2_/Si_3_N_4_/Cr/Au/ZnO floating thin-plate becomes more difficult, and the flatness of the thin-plate will decrease drastically. Figure 5a presents the scanning electron microscope (SEM) cross-sectional view and OM top view micrographs of the FPW floating thin-plate with the substrate silicon etched under 80 °C KOH solution. The floating thin-plate was deflected and deformed, resulting in peeling of some of the metal thin-film of the IDT, high insertion loss (>50 dB), and a low signal-to-noise ratio. Additionally, the deformed floating thin-plate was easily broken, resulting in low fabrication yield (<10%). Thus, conventional FPW devices manufactured using a high-temperature (80–90 °C) silicon etching process have limited applications. To overcome these disadvantages, we proposed a 60 °C (35.5 h)/27 °C (6.85 h) two-step low-temperature anisotropic wet etching process to solve the deformation and accurately control the thickness of the silicon floating thin-plate, which improved the fabrication yield and performance of the FPW transducer. A free-standing flat Si/SiO_2_/Si_3_N_4_/Cr/Au/ZnO floating thin-plate was obtained using the proposed etching process, as shown in Figure 5b, and the fabrication yield and performance of the FPW device were also improved.

**Figure 6:**
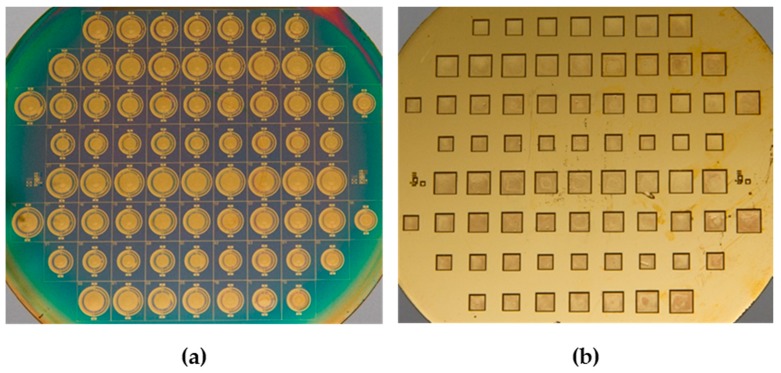
(**a**) Top-view and (**b**) bottom-view optical micrographs of the implemented FPW chip.

According to Semiconductor Manufacturing Technology, edited by M. Quirk and J. Serda [15], wafer fabrication yield is an important indicator of a wafer fab’s ability to produce a high-quality die. Yield is defined as the percent of good parts among the total initial group of parts. For example, as indicated by the top-view and bottom-view of the implemented wafer presented in Figure 6a,b, there were 72 FPW dies on the wafer, and 46 exhibited normal transducer performance, resulting in a fabrication yield of 63.88%. The fabrication yields of the other three FPW wafers in this study were 50%, 58.33%, and 86.11%, respectively, so the average wafer yield was 64.58%, which is higher than that of the conventional FPW device etched by high-temperature KOH solution.

### 4.2. Characterization of the Proposed FPW Devices

**Figure 7:**
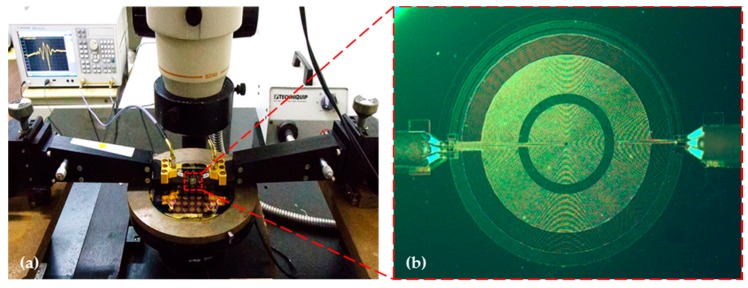
(**a**) On-chip measurement setup proposed in this study for characterization of the presented FPW devices; (**b**) Optical micrograph of the implemented FPW device with the circular IDTs/RGS configuration.

To characterize the developed FPW devices, we used an on-chip measurement setup consisting of a commercial Cascade RHM-06/V probe station and Agilent E5074 (Beaverton, OR, USA) network analyzer, as shown in Figure 7a. During the on-chip measurement (conducted at room temperature), as shown in Figure 7b, the 25/10 pairs of fan-shaped/circular input IDTs were contacted to the input Cascade ACP40-W coplanar 150 Ground-Signal-Ground (GSG) probes, and the insertion loss and operating frequency of the FPW devices were extracted by the output GSG probes.

**Figure 8:**
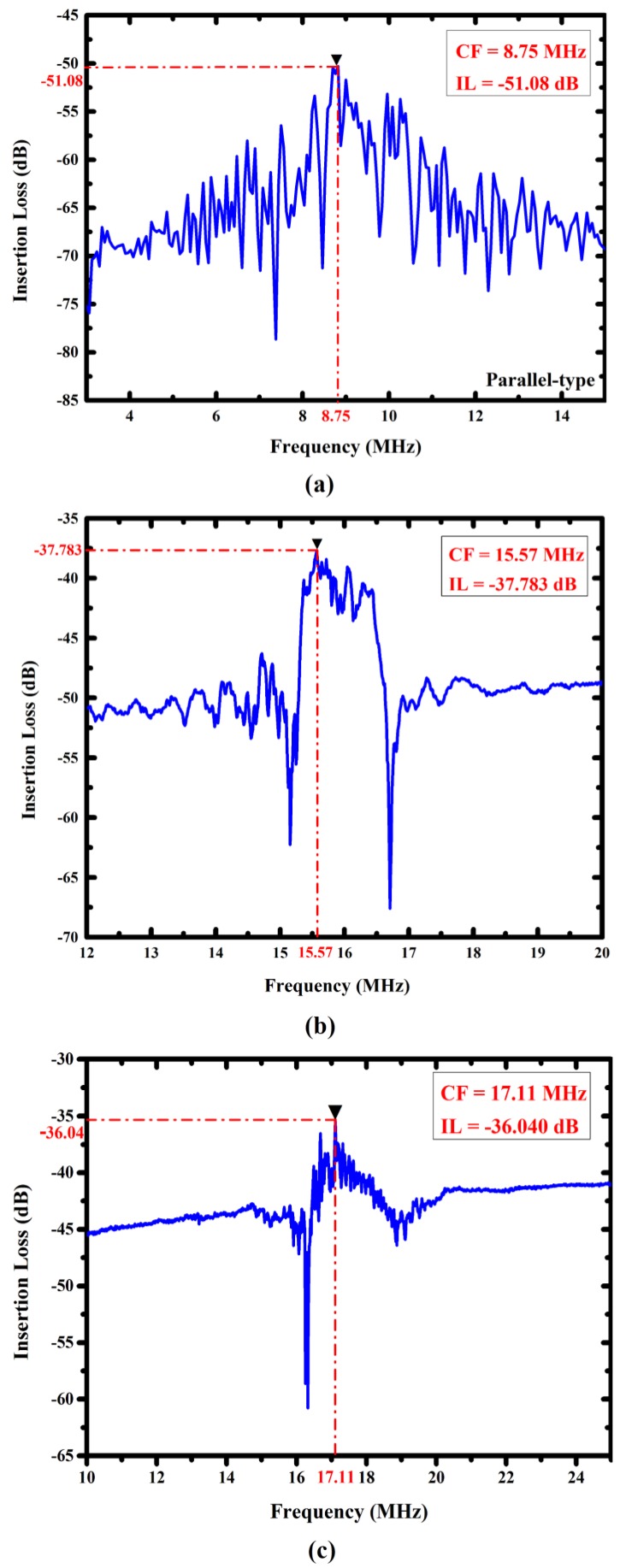
Frequency responses of the FPW device with (**a**) conventional parallel type; (**b**) fan-shaped type; and (**c**) circular type IDTs/RGS configurations.

Our previous study [7] presented an FPW-IgE biosensor whose frequency response is shown in Figure 8a. Although the center frequency of this device was only 8.75 MHz, its insertion loss was high (−51.08 dB), and its signal-to-noise ratio was low because the RGS configuration was not adopted and the IDT geometry was the conventional parallel type. In this study, fan-shaped and circular IDTs/RGS configurations of FPW devices were proposed to solve these disadvantages. As shown in Figure 8b,c, both types of FPW devices have low insertion loss (−37.78 dB for fan-shaped IDTs/RGS and −36.04 dB for circular IDTs/RGS), and their signal-to-noise ratios were higher than that of the conventional FPW device. Both the fan-shaped and circular IDTs/RGS configurations can increase the wave density, improving the efficiency of the launched flexural plate-wave propagated from the left-side input IDTs to the right-side output IDTs. Additionally, compared with the fan-shaped IDTs/RGS, the circular IDTs/RGS had slightly lower insertion loss, because the edge effect of the electric field was completely eliminated.

The IDTs/RGS geometry, silicon etching parameters, and FPW characteristics of the proposed focus-type and conventional FPW transducers are compared in Table 2. The design specifications of the floating thin-plate (silicon or silicon dioxide) from the three studies were 3-μm Si, 5-μm SiO_2_ and 15-μm Si, respectively. The total thickness of the floating thin-plate was 5.29 ± 3.5 μm, 6.32 ± 5 μm and 16.86 ± 2 μm, respectively, including the other insulator/metal/piezoelectric thin-film. The deviation in the thickness of the silicon thin-plate and the fabrication yield of the FPW devices were affected by the anisotropic wet etching temperature. In our two previous studies, a thickness deviation of 3.5–5 μm was approached based on the design specification of the silicon thickness, resulting in a very low wafer fabrication yield (<15%). Through the low-temperature two-step etching process, the silicon thickness deviation was reduced to 2 μm, and the wafer yield was improved to 50%–86.11%. Furthermore, compared with conventional FPW devices, the two proposed focus-type FPW devices also had lower insertion loss (−37.78 dB for fan-shaped and −36.04 dB for circular).

### 4.3. Characterization of the Proposed FPW-CEA Biosensor

**Figure 9:**
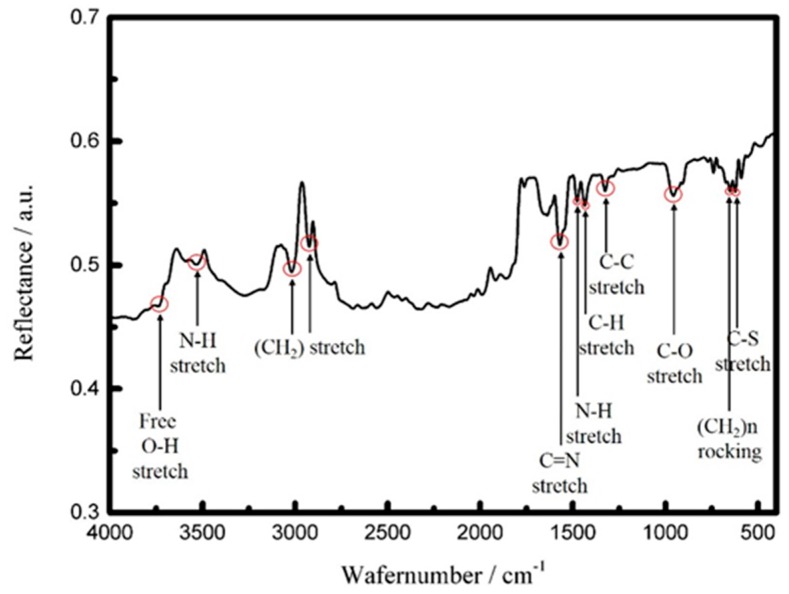
FT-IR spectrum of cystamine SAM/glutaraldehyde cross-linking layers.

An FT-IR (70 v/s, Bruker, Billerica, MA, USA) spectroscopy system was used to investigate and analyze the structural perturbation changes of cystamine SAM/glutaraldehyde molecules. The background spectra were recorded at a resolution of 4 cm^−1^ over 512 scans in the frequency region 400−4000 cm^−1^ at room temperature. The measurements were corrected based on a silicon wafer deposited with thin Cr/Au metal layers as a reference background. The measured FT-IR spectrum of the cystamine SAM/glutaraldehyde layers is shown in Figure 9. The important peaks in the spectrum represent the C–S stretch, (CH_2_)n rocking, C–O stretch, C–C stretch, C–H stretch, N–H bend, C=N stretch, CH_2_ stretch, N–H stretch, and free O–H stretch. These peaks appear at 622.96, 650.93, 955.65, 1325.96, 1434.93, 1477.36, 1569.94, 2922.9, or 3018.37, 3531.39, and 3738.72 cm^−1^, respectively. The absorption bands in the FT-IR spectrum agree with the structural formula of the cystamine SAM/glutaraldehyde layers and further confirm the surface modification of the gold-coated substrate with the cystamine self-assembled monolayer.

**Figure 10:**
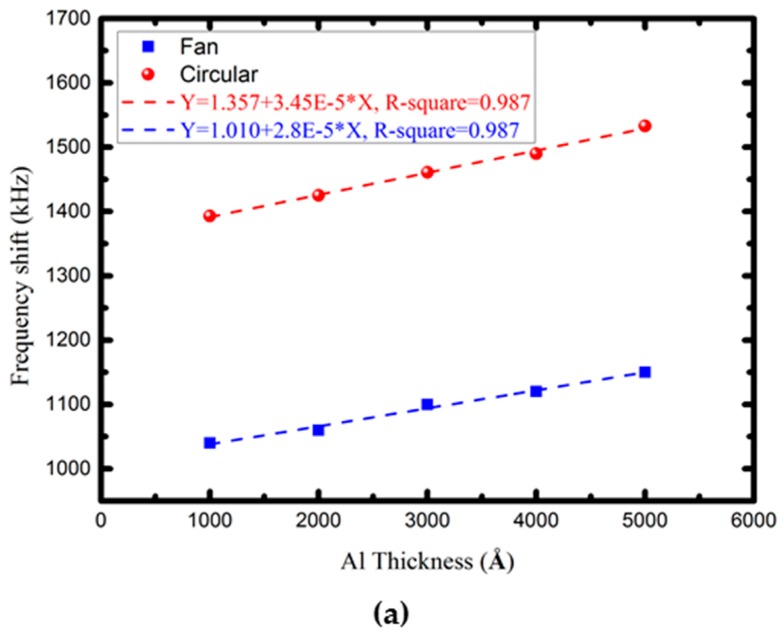

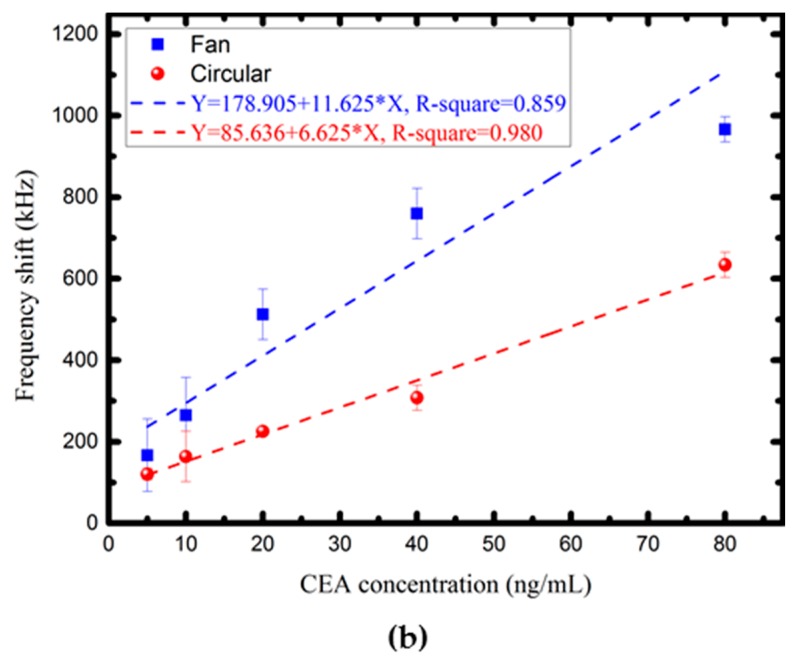
Measured frequency shift of (**a**) the FPW device under five different Al mass loadings and (**b**) the FPW-CEA biosensor under five different CEA antigen concentrations.

To analyze the solid-state mass sensitivity of the proposed focus-type FPW transducers, the frequency was measured before and after five different thicknesses of Al thin-film (from 1000 Å to 5000 Å, with a pitch thickness of 1000 Å) were deposited onto the surface of the backside silicon. As depicted in Figure 10a, at the five different Al masses (from 27 μg/cm^2^ to 135 μg/cm^2^ at intervals of 27 μg/cm^2^), the frequency shifts of the fan-shaped and circular FPW devices were (1.04, 1.06, 1.1, 1.12, and 1.15 MHz) and (1.39, 1.43, 1.46, 1.49, and 1.53 MHz), respectively. As calculated according to Equation (1), the mass sensitivities of the proposed FPW devices with fan-shaped IDTs/RGS and circular IDTs/RGS were 60.16 cm^2^/g and 70.06 cm^2^/g, respectively. The two proposed FPW transducers had identical high sensing linearity (R-square = 0.987), which is beneficial for developing the readout circuit of such a device.

After the cystamine SAM/glutaraldehyde/CEA antibody multilayer was deposited on the backside Cr/Au, the center frequency shift was measured under five different concentrations of the CEA antigen (5, 10, 20, 40, and 80 ng/mL) coated on the backside cavity of the FPW-based CEA biosensor. Each testing condition was measured three times. Compared with the center frequency before CEA antigen coating (18.78 MHz for fan-shaped and 17.44 MHz for circular), the measured frequency shifts of the fan-shaped and circular FPW were (167, 265.07, 512.6, 760.07, and 966.37 kHz) and (120, 163.67, 225.53, 308.07, and 634 kHz), respectively. As shown in Figure 10b, both proposed FPW-based CEA biosensors have low detection limits for the CEA antigen (5 ng/mL), whereas the FPW transducer with the circular type IDTs/RGS has very high sensing linearity (R-square = 0.98). Figure 2a presents that the fan-shaped IDTs/RGS does not cover the whole central area of the floating thin-plate, so some of the cystamine SAM/glutaraldehyde/CEA antibody/CEA antigen mass loaded onto the backside surface probably was not sensed, resulting in a lower sensing linearity in comparison with that of the FPW device with a circular IDTs/RGS configuration.

## 5. Conclusions

To reduce insertion loss (−40.85 to −51.08 dB) and enhance the fabrication yield (<15%) of conventional FPW devices, two novel focus-type IDTs/RGS configurations and a two-step low-temperature (60 °C/27 °C) anisotropic silicon etching process were proposed in this study. Both types of the implemented FPW devices had low insertion loss (−36.04 to −37.78 dB), high fabrication yield (50%–86.11%), and low center frequency (15.57–17.11 MHz), which can facilitate the development of a readout IC and bio-sensing microsystem. Additionally, a novel FPW-based CEA biosensor was further developed using microelectromechanical systems (MEMS) and self-assembly monolayer technologies. The realized FPW-CEA biosensors had a low detection limit for CEA antigen (5 ng/mL), short response time (<5 min), high sensing linearity (R-square = 0.98), and moderate mass-sensitivity (60.16 cm^2^/g for fan-shaped IDTs/RGS and 70.06 cm^2^/g for circular IDTs/RGS).

## Figures and Tables

**Table 1:** Design specifications of the fan-shaped and circular IDTs/RGS configurations.

Parameter	Value
IDTs finger width/gap	25 μm/25 μm
Wavelength of IDTs	100 μm
Number of IDTs’ finger pairs	10
RGS pairs	10
RGS finger width/gap	25 μm/25 μm
Separation between the RGS and IDTs	37.5 μm

**Table 2:** Comparison of the IDTs/RGS geometry, silicon etching parameters, and FPW characteristics of the proposed focus-type and conventional FPW transducers [7,8].

References	IDTs/RGS Geometry	Temperature of the Si Etching Process (°C)	Thickness of the Floating Thin-Plate (m)	Insertion Loss (dB)	Fabrication Yield (%)
I-Yu Huang [7]	Parallel (without RGS)	60 (one-step)	5.29 ± 3.5	−51.08	<10
Chang-Yu Lin [8]	Parallel (with RGS)	80 (one-step)	6.32 ± 5	−40.85	10–15
This Study	Fan-shaped (with RGS)	60/27 (two-step)	16.86 ± 2	−37.78	50–86.11
	Circular (with RGS)			−36.04	
